# Sustainable Natural Product Glycosylation: A Critical Evaluation of Biocatalytic and Chemical Approaches

**DOI:** 10.1002/cssc.202501094

**Published:** 2025-09-15

**Authors:** Felipe Mejia‐Otalvaro, Brianna Marie Lax, Onur Kırtel, Ditte Hededam Welner

**Affiliations:** ^1^ The Novo Nordisk Center for Biosustainability Technical University of Denmark Søltofts Plads Building 22 Kgs. Lyngby DK‐2800 Denmark

**Keywords:** biocatalysis, chemical catalysis, glycosylation, natural products, sustainability

## Abstract

The glycosylation of natural products can significantly enhance their physicochemical properties, with numerous synthetic and biocatalytic methodologies continuously being developed, each presenting unique advantages and challenges. Biocatalytic methods are often presumed to be more sustainable alternatives to chemical approaches; however, their environmental and economic viabilities require critical evaluation. This review summarizes the recent advancements in natural product glycosylation and provides a comprehensive techno‐economic and environmental assessment based on yield, titer, rate, environmental factor, and impact on endpoint categories using a life cycle impact assessment approach. Although biocatalytic methods are highlighted for their superior yields, they are frequently hindered by lower titers and reaction rates compared to their chemical counterparts. Surprisingly, chemical glycosylation exhibited lower environmental factors (E‐factors), whereas biocatalytic approaches displayed lower impacts on endpoint categories, highlighting that E‐factors fail to capture the environmental implications of a process. This review demonstrates the challenges associated with quantifying the environmental impacts of a process, especially given the lack of experimental detail reporting in the biocatalytic field. It exposes the misguided assumption that biocatalytic processes always exert lower environmental burdens and identifies key opportunities to enhance process efficiency and sustainability, providing guidance for selecting and developing a given natural product glycosylation reaction.

## Graphical abstract

Developing a process to produce glycosylated natural products involves many decisions, and given the current climate crisis, the environmental impact of the various processes must be a major consideration. This review provides an overview of the various processes and a framework for which metrics should be quantified to select processes that are both economically viable and sustainable. LCA: Life‐cycle assessment.

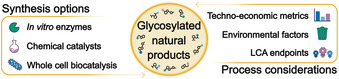

## 1 Introduction

Natural products (NPs) include primary and secondary metabolites produced in living organisms that serve essential functions and confer additional fitness, respectively. Many NPs have found applications as industrial chemicals, glycosylated either in their endogenous forms or during their commercialization due to the enhanced biochemical and functional properties that glycosylation affords.^[^ ^1^, ^2^ ^]^ The applications of glycosylated NPs and their derivatives include compounds used in the pharmaceutical, agriculture, cosmetic, and food sectors, among others.^[^ ^3^, ^4^, ^5^ ^]^ In light of the breaching of several planetary boundaries, there is an urgent need for sustainable processes,^[^ ^6^ ^]^ and replacing the traditional chemical extraction of glycosylated NPs from plants with biocatalytic methods as a means to do so is fueled by the school of thought that biocatalytic processes are lower impact than their chemical counterparts. There are many reviews on NP glycosylation, including advancements in glycosylation chemistry,^[^ ^7^, ^8^ ^]^ the role of biocatalysis in the industry,^[^ ^9^ ^]^ and the utilization of glycosylating enzymes.^[^ ^10^, ^11^, ^12^ ^]^

However, there is a concerning gap in the literature regarding the comparison of the environmental impacts of these various approaches. There are a number of important quantifiable techno‐economic and environmental metrics that can be utilized for determining the viability and sustainability of an industrial process (**Table** **1**),^[^ ^13^, ^14^, ^15^, ^16^, ^17^ ^]^ but they have not been applied rigorously for assessing which processes would actually exhibit better environmental performances, with the exception of a thorough analysis of the efficiency of nucleoside glycosylation routes.^[^ ^18^ ^]^ There are a handful of recent reviews that highlight general approaches for improving the sustainability of biocatalytic processes, but these do not consider the specifics of glycosylation reactions.^[^ ^19^, ^20^, ^21^, ^22^ ^]^ Thus, the aim of this review is to (1) summarize the advancements in NP glycosylation from the past three years and (2) compare the relative impacts of these approaches to more systematically determine how to improve the environmental performances of NP glycosylation methods. Of note, this review only covers the glycosylation of small molecules, not including sugars, proteins, or nucleosides.

**Table 1:** Utilized techno‐economic and environmental metrics to assess the sustainability of NPs glycosylation.

Metric	Definition	Equation
Titer (mM)	Product concentration after the glycosylation reaction.^[^ ^14^, ^16^ ^]^	$\text{Titer = }\frac{\text{moles of product}}{\text{volume of reaction}}$
Yield (%)a)	Moles of product per moles of substrate.^[^ ^14^, ^16^ ^]^	$\text{Yield = }\frac{\text{moles of product}}{\text{moles of substrate }}\text{×100}$
Rate (mM.h^−1^)	Productivity of the reaction.^[^ ^14^, ^16^ ^]^	$\text{Rate = }\frac{\text{product concentration}}{\text{reaction time }}$
Environmental factor (E‐factor)	E‐factor considers the mass of waste produced per mass of product.	E‐$\text{factor = }\frac{\text{total mass of waste}}{\text{mass of product }}$
Ecosystem quality (Species.year^−1^)	Biodiversity loss measured as the number of species lost per year.^[^ ^161^ ^]^	ReCiPe 2016 Endpoint (H) methodology using ecoinvent v3.8 database and open LCA 2.1.^[^ ^161^ ^]^
Human health (DALY)	Disability‐adjusted life years (the loss corresponding to years of full health).^[^ ^161^ ^]^	
Resource scarcity	Surplus cost of future production quantified in USD to the year 2013.^[^ ^161^ ^]^	

a) For the chemical methods, nonisolated yields were used where reported; isolated yields were used otherwise.

## 2 Recent Advancements in Glycosylation Reactions

This section provides a summary of the advancements in glycosylation reactions from the past three years. These advancements are divided into four categories: chemical glycosylation, in vitro biocatalytic glycosylation, in vivo glycosylation (whole cell and de novo syntheses), and novel approaches to improve the sustainability of these glycosylation reactions.

### 2.1 Chemical Glycosylation

The advent of chemical glycosidic bond formation, initiated with the Fischer reaction and expanding to include stereochemical control with the Koenigs–Knorr reaction, revolutionized carbohydrate synthesis.^[^ ^23^, ^24^ ^]^ These traditional chemical methods rely on hydroxyl protecting and deprotecting strategies to achieve the regio and stereoselective glycosylation that is usually required for the applications of glycosylated NPs. These lengthy syntheses often result in complicated processes with harsh conditions, extensive purifications, and large amounts of waste generation.^[^ ^25^, ^26^ ^]^ To combat such issues, improved chemical methodologies that retain the desired selectivity began with the development of one‐pot reactions^[^ ^27^ ^]^ and solid‐supported synthesis.^[^ ^28^ ^]^ In the past three years, improvements to the chemical glycosylation field have continued to build upon these pioneering approaches and have also expanded to include new chemistries that have greater inherent stereochemical control. One‐pot domino reactions are convenient because all reactants for all steps are added together at the beginning, and the full syntheses proceed one step after another without any intervention. These types of reactions continue to show great success at reducing the number of reaction steps and required reagents for some carbohydrate chemistries.^[^ ^29^ ^]^ Condensing a complex reaction into a one‐pot domino reaction can provide significant benefits from a materials perspective, especially in reducing solvent use and removing the need for purification of intermediate compounds. The most recent advancements on this front include a ruthenium (Ru)‐catalyzed single transformation for *meta*‐*C*‐alkyl glycosides,^[^ ^30^ ^]^ a one‐step glycosylation reaction of flavonoids from unprotected precursors,^[^ ^31^ ^]^ and a single‐pot reaction for combined donor functionalization and *C*‐glycosylation.^[^ ^32^ ^]^ Other efforts at reducing the burden of chemical carbohydrate synthesis include process and catalyst optimization^[^ ^33^, ^34^, ^35^ ^]^ and the development of bench‐stable donors for convenient glycosylation.^[^ ^36^ ^]^

**Figure 1:**
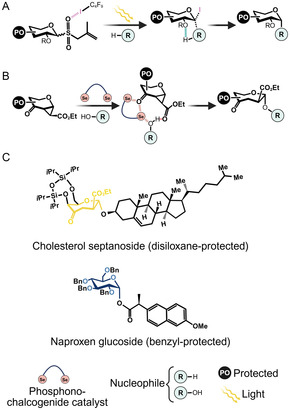
Examples of how noncovalent interactions between the donor and acceptor and catalyst are used in synthetic glycosylation chemistry. Noncovalent interactions are shown as dashed lines. A) Example of noncovalent interactions in a photoinducible glycosylation reaction. A halogen bond‐activated donor undergoes radical generation using light, followed by hydrogen bond‐assisted nucleophilic substitution. Schematic adapted from [40]. B) Example of noncovalent interactions in a chalcogen‐bonding catalyst. Acceptor positioning is controlled via chalcogen and hydrogen bonding, resulting in a stereoselective nucleophilic substitution. Schematic adapted from [45]. C) Examples of NPs that are glycosylated using these methods.

Many of the new methodologies focus on either novel donor functionalization strategies or catalysts, which represent two of the most important aspects of glycosylation chemistry. An important trend in both donor functionalization and catalyst development is the manipulation of noncovalent interactions between the donor and the catalyst, which promote important conformational changes that imbue these catalysts with superior regio and stereoselectivities (**Figure** **1**). These noncovalent interactions are inspired by those that provide stereochemical control in the active sites of enzymes and have become a staple of modern carbohydrate chemistry.^[^ ^37^, ^38^ ^]^ Noncovalent interactions can be found as crucial components for many glycosylation chemistries, including radical‐mediated aminoboronic catalysis, in which hydrogen bonding facilitates the required proximity of the radical intermediate and the acceptor,^[^ ^39^ ^]^ and halogen‐bond‐assisted radical‐mediated glycosylation, where halogen bonding promotes donor activation and hydrogen bonding provides stereochemical control (Figure 1A).^[^ ^40^ ^]^ One interesting use of noncovalent interactions for donor functionalization is a glycosylation reaction that is initiated by a noncovalent chelation process for donor activation using a scandium catalyst; however, this method was not used for the glycosylation of any small molecule NPs.^[^ ^41^ ^]^ Noncovalent catalysts have shown impressive selectivities, and they come in a variety of formats, including thiourea catalysts,^[^ ^42^, ^43^ ^]^ halogen bonding catalysts,^[^ ^40^ ^]^ and chalcogen‐bonding catalysts,^[^ ^44^, ^45^, ^46^, ^47^, ^48^ ^]^ among others.^[^ ^49^ ^]^ One impressive study shows the ability of bis‐thiourea small molecule catalysts to *O*‐glycosylate unprotected or minimally protected sugars, although sugar glycosylation is not the focus of this review.^[^ ^42^ ^]^ The phosphonoselenide catalysts, part of the chalcogen‐bonding catalyst family, take advantage of noncovalent interactions to induce conformational changes in the sugar donors that result in the impressive stereochemical control of these reactions (Figure 1B).^[^ ^44^, ^45^ ^]^

**Figure 2:**
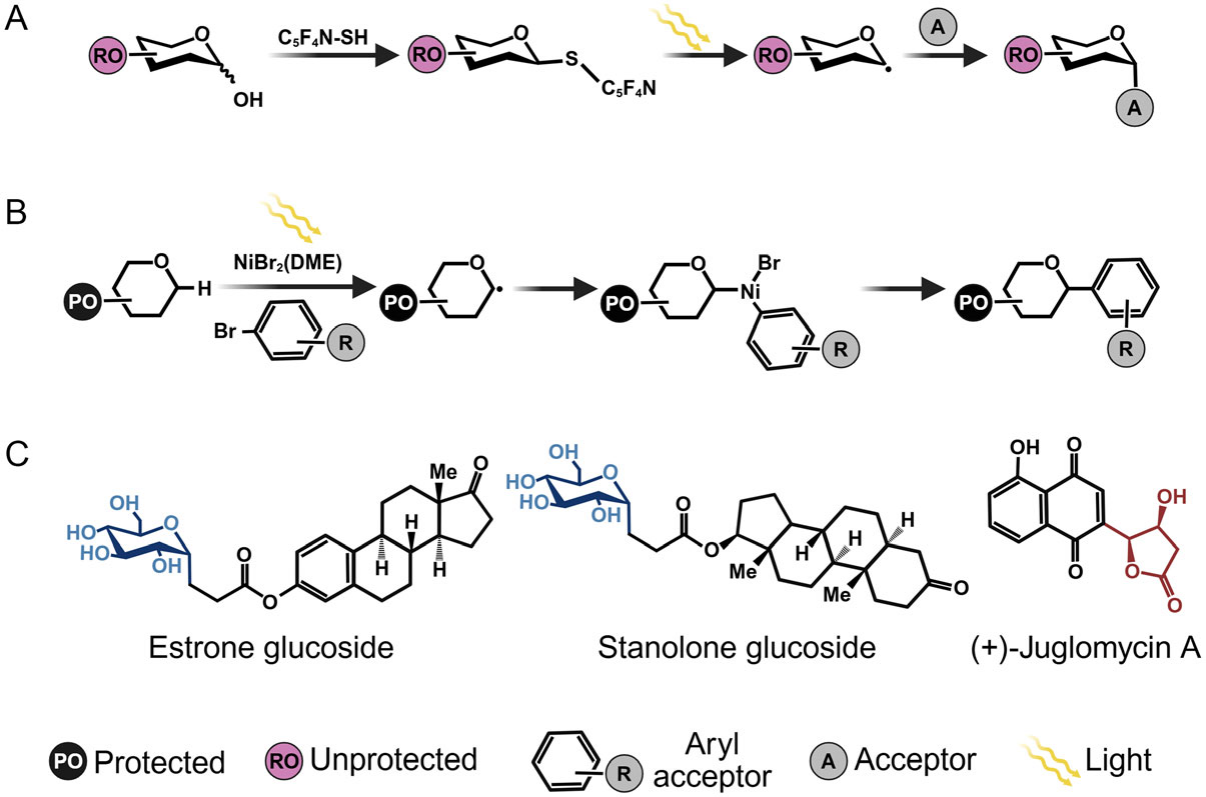
Examples of radical‐mediated synthetic glycosylation chemistry. Radicals are denoted by single dots. A) Example of radical‐mediated photoinducible glycosylation, beginning with the preparation of the required donor and followed by radical generation with light and nucleophilic substitution. Schematic adapted from [53]. B) Example of radical‐mediated photometallo‐catalyzed glycosylation. The donor radical is first generated using light, followed by radical recombination and reductive elimination to yield the final glycoside. Schematic adapted from [56]. C) Examples of NPs that are glycosylated using these methods.

Replacing ionic glycosylation with radical‐mediated approaches has also become one of the biggest trends in modern synthetic glycosylation (**Figure** **2**).^[^ ^50^, ^51^ ^]^ Radical‐mediated glycosylation can generally alleviate issues with glycosyl precursor preparation, specifically protecting chemistry, since it has a greater tolerance for functional groups and can be used in the glycosylation of complex molecules. Additionally, radical‐mediated reactions often use milder conditions since they do not require strong acids for donor activation like ionic methods do. Rather, they can use photoredox‐, thermal‐, or metal‐based activation strategies. Photoinducible glycosylation is the most commonly used radical‐mediated approach, which utilizes light to generate radicals,^[^ ^8^ ^]^ and there are numerous reports of novel photoinducible glycosylation chemistries that occur under mild conditions with reusable catalysts.^[^ ^39^, ^40^, ^52^, ^53^, ^54^, ^55^, ^56^, ^57^, ^58^ ^]^ One of the most exciting reports is the direct functionalization of unprotected sugars via radical‐based cross‐coupling to the compound of interest in the presence of light (Figure 2A).^[^ ^53^ ^]^ Other photocatalytic approaches that remove or lessen the requirement of protecting groups on the donor include the use of aminoboronic catalysts^[^ ^39^ ^]^ and nickel‐catalyzed cross‐coupling for *C*‐glycosylation (Figure 2B).^[^ ^56^ ^]^ Catalyst‐free photoinducible glycosylation reactions include intramolecular proton transfer between the donor and acceptor^[^ ^59^ ^]^ and a glycosylation‐cyclization cascade.^[^ ^60^ ^]^ Other recent radical‐mediated approaches include electrochemical donor activation via halogen‐atom transfer,^[^ ^61^ ^]^ *C*‐aryl cross‐coupling via nickel or iron catalysts,^[^ ^62^ ^]^ and dehydroxylative donor activation coupled with metallaphotoredox catalysis,^[^ ^54^ ^]^ although these still require the use of protecting groups.

**Figure 3:**
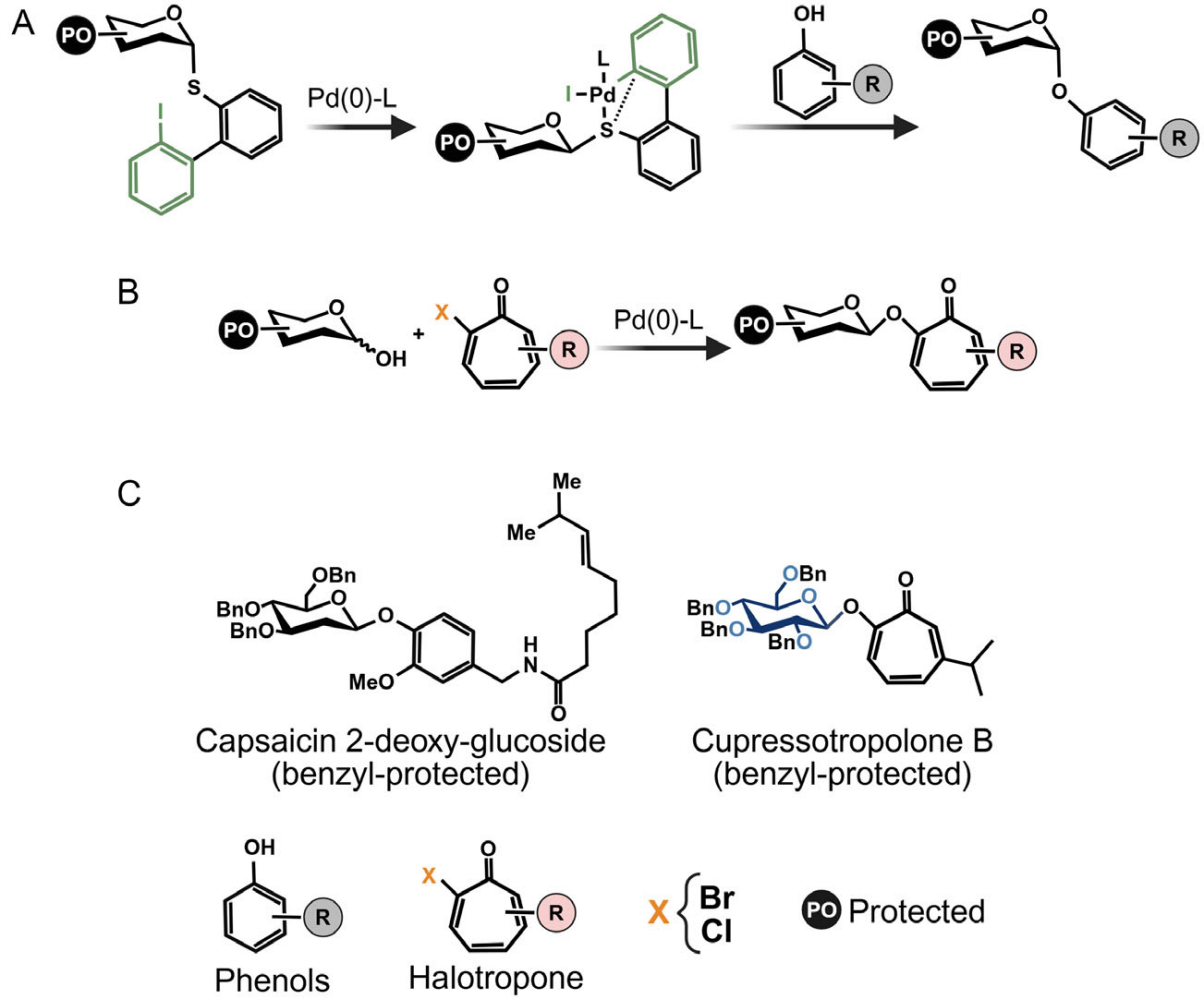
Examples of transition‐metal catalysts used in synthetic glycosylation chemistry. A) Example of Pd‐catalyzed glycosylation reaction. The glycosyl donor undergoes oxidative addition with the Pd catalyst, which undergoes nucleophilic attack by the acceptor, resulting in the final glycoside. Schematic adapted from [63]. B) Example of Pd‐catalyzed base‐promoted glycosylation reaction. The halotropone undergoes ipso‐substitution for the coupling of the final glycoside. Schematic adapted from [66].

Transition‐metal catalysts have also emerged as efficient and selective solutions for glycosylation reactions that remain challenging for traditional approaches (**Figure** **3**).^[^ ^26^ ^]^ A recent landmark study showing palladium (Pd)‐catalyzed bimolecular nucleophilic substitution (S_N_2) for the *O*‐glycosylation of phenols has revolutionized the role of Pd catalysts in contemporary carbohydrate chemistry (Figure 3A).^[^ ^63^ ^]^ This elegant approach utilizes an iodobiphenyl‐substituted sulfide as a glycosyl donor, which once complexed with the Pd catalyst, undergoes nucleophilic attack by the acceptor via an S_N_2 mechanism, providing excellent stereochemical control. This method shows impressive donor and acceptor scopes and also demonstrates the glycosylation of NP derivatives in one‐pot reactions.^[^ ^63^ ^]^ Many other recent reports also use Pd catalysts for novel *C*‐glycosylation reactions, including for alkynylanilines,^[^ ^64^ ^]^ *sp* ^*3*^‐hybridized carbon atoms, mostly in sugars,^[^ ^65^ ^]^ halogenated tropones (Figure 3B),^[^ ^66^ ^]^ and iodoglycals for the synthesis of oligosaccharides.^[^ ^67^ ^]^ Pd catalysts have also been used for *N*‐*O*‐glycosylation of oximes,^[^ ^68^, ^69^ ^]^ *O*‐glycosylation of aromatic compounds^[^ ^70^ ^]^ and NPs,^[^ ^71^ ^]^ and *S*‐glycosylation for a variety of thiols.^[^ ^72^ ^]^ Although the most common transition‐metal catalysts are Pd‐based, there are a few reports showing the use of other transition metals, including various Ru catalysts,^[^ ^30^, ^73^, ^74^ ^]^ radical‐mediated nickel or iron catalysts,^[^ ^62^ ^]^ and cobalt, copper, and gold catalysts.^[^ ^75^, ^76^, ^77^, ^78^ ^]^ One study shows the ability of titanium catalysts to perform radical *C*‐glycosylation using unprotected sugars, including a few examples of natural product derivatives.^[^ ^79^ ^]^

Lastly, there are a few new glycosylation reactions that use nonconventional methodologies for advancing contemporary carbohydrate synthesis. The use of calcium‐based catalysts for *N*‐*O*‐glycosylation of oximes with unprotected donors showed good selectivity and scalability.^[^ ^80^ ^]^ Another study showed the ability to replace metal‐based catalysts with I_2_ for donor activation using benzoate donors.^[^ ^81^ ^]^ One catalyst‐free approach is able to perform *C*‐glycosylation with excellent stereochemical control via functional group deletion and rearrangement that yields the final glycosylated product.^[^ ^82^ ^]^

### 2.2 In Vitro Biocatalytic Glycosylation

In vitro biocatalytic glycosylation of NPs has been mostly focused on the implementation of glycosyl hydrolases (GHs) and the uridine diphosphate‐dependent glycosyltransferase (UGT)‐sucrose synthase (SuSy) cascade. Each enzymatic system offers distinct advantages and limitations, and recent advances have aimed to expand the substrate scope and enhance efficiencies through enzyme engineering.

**Figure 4:**
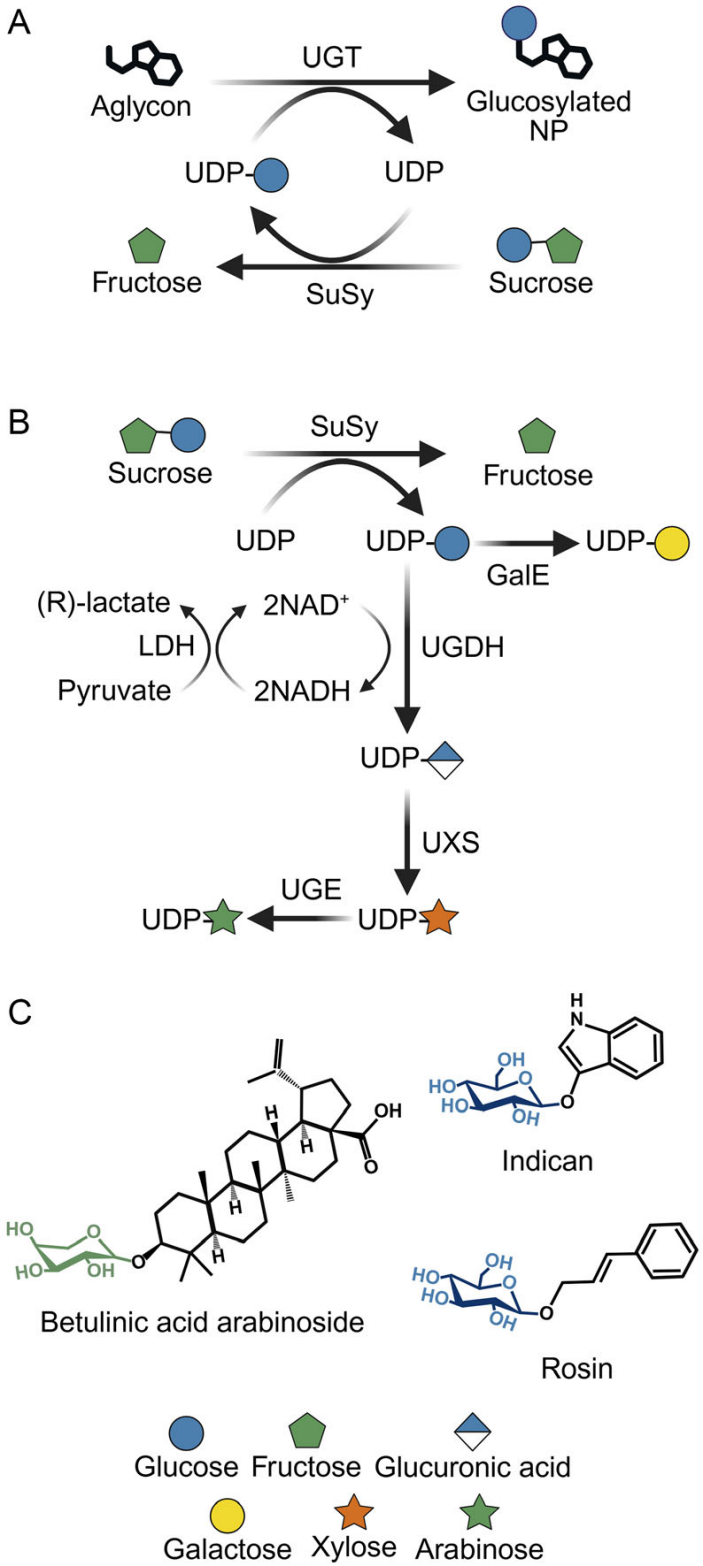
Examples of UGT‐SuSy catalyzed glycosylation reactions. A) Schematic reaction of in vitro glycosylation of NPs using the UGT‐SuSy cascade. B) Biocatalytic synthesis of different UDP‐sugar donors from UDP and sucrose.^[^ ^84^, ^89^, ^90^ ^]^ C) Examples of glycosylated NPs reported to be glycosylated by the UGT‐SuSy cascade.^[^ ^84^, ^93^, ^166^ ^]^ GalE: UDP‐galactose‐4‐epimerase, UGDH: UDP‐glucose 6‐dehydrogenase, UXS: UDP‐xylose synthase, UGE: UDP‐glucose 4‐epimerase, and LDH: lactate dehydrogenase.

UGTs catalyze the transfer of a sugar group from a nucleotide‐activated donor to an NP acceptor (**Figure** **4**A). UGTs perform *β*‐glycosylation using an inverting mechanism to form glycosidic bonds, but these enzymes often suffer from substrate inhibition and dilution‐induced inactivation, thus requiring optimization before biotechnological implementation.^[^ ^83^ ^]^ In vitro UGT‐SuSy cascades offer a cost‐effective strategy for NP glycosylation by recycling uridine diphosphate glucose (UDP‐Glc), whose further conversion to other UDP‐glycosyl donors (Figure 4B) enables the production of a wide range of glycosides (Figure 4C).^[^ ^84^, ^85^, ^86^, ^87^, ^88^, ^89^, ^90^, ^91^ ^]^ For instance, arabinosylated betulinic acid was synthesized in a one‐pot, five‐enzyme cascade reaction. In this system, *Gu*SuSy catalyzed the formation of UDP‐Glc, which was subsequently converted to UDP‐Arabinose (UDP‐Ara) by UDP‐glucose 6‐dehydrogenase (UGDH), UDP‐xylose synthase (UXS), and UDP‐Glc 4‐epimerase (UGE) (Figure 4B). In the final step, UGT99D1 transferred the arabinose moiety to betulinic acid, releasing UDP and starting a new sugar donor recycling cycle.^[^ ^84^ ^]^

Enzyme engineering has played a crucial role in improving product yields and enhancing enzyme robustness for industrial applications. Improved catalytic efficiencies of UGTs have been achieved via semi‐rational enzyme engineering approaches, including the combination of substrate docking, alanine scanning, and saturation mutagenesis.^[^ ^85^, ^86^, ^87^, ^88^, ^92^ ^]^ This approach enhanced substrate binding and transport of the bacterial glycosyltransferase YojK, resulting in a 7.35‐fold increase in catalytic activity and enabling the scale production of the sweetener Rebaudioside D.^[^ ^86^ ^]^ In another example, stability engineering was employed to *Pt*UGT1, which yielded a thermostable variant with a 13.1 °C increase in melting temperature, enabling the synthesis of 65 mM indican and a more sustainable denim dyeing process.^[^ ^93^ ^]^

The use of SuSy was essential to develop these glycosylation reactions, as it drives the reaction forward using minimal UDP concentrations and prevents UDP‐mediated inhibition of UGT activity. Despite this essential role of SuSy, only a few recent studies address the enhancement of SuSy stability and activity. These include the identification of *Micractinium conductrix* SuSy (*Mc*SuSy),^[^ ^94^ ^]^ the mutation of the *N*‐terminal serine phosphorylation site to enhance activity,^[^ ^84^, ^94^, ^95^ ^]^ and the engineering of *Nitrosospira multiformis* SuSy (*Nm*SuSy) for improved stability and activity.^[^ ^96^, ^97^, ^98^ ^]^ However, the limited diversity of SuSys currently employed could be constraining the development of efficient and versatile UGT‐SuSy glycosylation systems, and a more robust SuSy variant with high catalytic efficiency and thermo‐ and chemo‐stabilities remains necessary.

**Figure 5:**
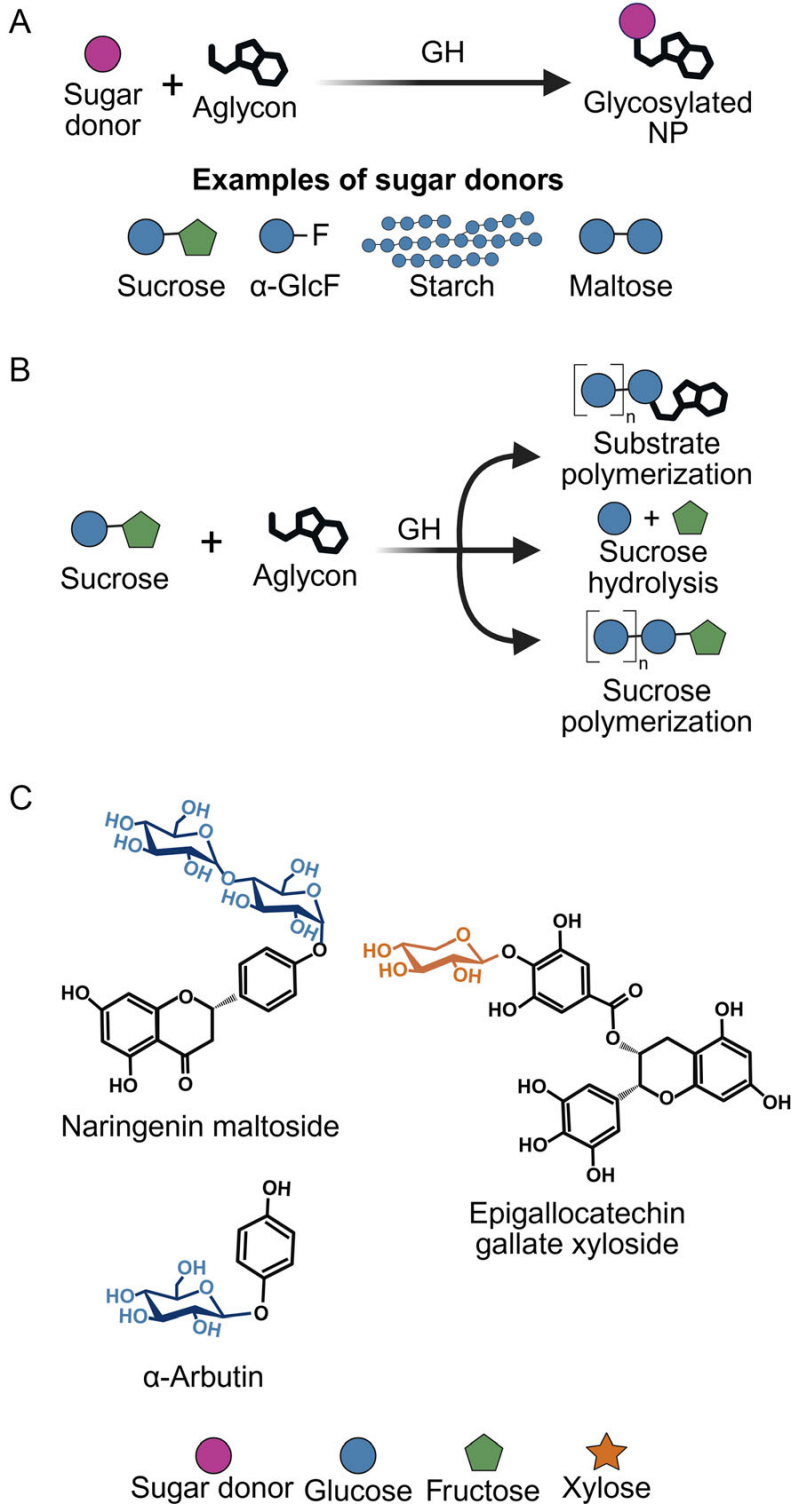
Examples of GH‐catalyzed glycosylation reactions. A) Schematic reaction for the in vitro glycosylation of NPs using the GHs with various sugar donors, including α‐glucose fluoride (α.‐GlcF).^[^ ^101^, ^106^, ^108^, ^110^ ^]^ B) Competing side activities that hinder GHs mediated glycosylation, such as sucrose hydrolysis and polymerization, leading to undesired polymerized glycosides.^[^ ^99^, ^100^, ^108^ ^]^ C) Examples of NPs reported to be glycosylated by GHs.^[^ ^103^, ^110^, ^111^ ^]^

In contrast to UGTs, GHs can glycosylate NPs in vitro by transglycosylation, transferring a sugar moiety directly from substrates such as sucrose, maltose, or starch. This bypasses the requirement for sugar nucleotides, representing a direct advantage over the UGT‐SuSy cascade (**Figure** **5**A). Enzymes such as cyclodextrin glycosyltransferases (GH13, EC 2.4.1.19), glucansucrases (GH70, EC 2.4.1.5), branching sucrases (GH70, EC 2.4.1.362/373), amylosucrases (GH13, 2.4.1.4), levansucrases (GH68, EC 2.4.1.10), and sucrose phosphorylases (GH13, EC 2.4.1.7) exhibit high transglycosylation activity on sugar substrates. However, their activity on nonsugar substrates is hindered by competing polymerizing and hydrolytic activities (Figure 5B). These competing activities reduce sugar donor availability, directly compromising process feasibility. Additionally, polymerizing activities often result in a mixture of glycosides rather than a single product, hampering reproducibility and downstream processing, as well as the targeted application of the glycosylated NPs (Figure 5B).^[^ ^99^, ^100^ ^]^ Despite these challenges, several NPs have been reported to be glycosylated by GHs, including flavonoids and terpenoids such as α‐arbutin, genistein, naringenin, ganoderic acids, and catechins (Figure 5C).^[^ ^101^, ^102^, ^103^, ^104^, ^105^, ^106^ ^]^ Additionally, enzyme engineering strategies have been employed to improve yields and efficiencies. These strategies include enhancing acceptor affinity,^[^ ^108^ ^]^ modifying the nucleophile of glucosidases to obtain glycoligase and glycosynthase activities,^[^ ^99^, ^109^, ^110^ ^]^ and using glycosidase treatment to eliminate polymerized chains, yielding monoglucosides.^[^ ^111^ ^]^

### 2.3 In Vivo Biocatalytic Glycosylation

**Figure 6:**
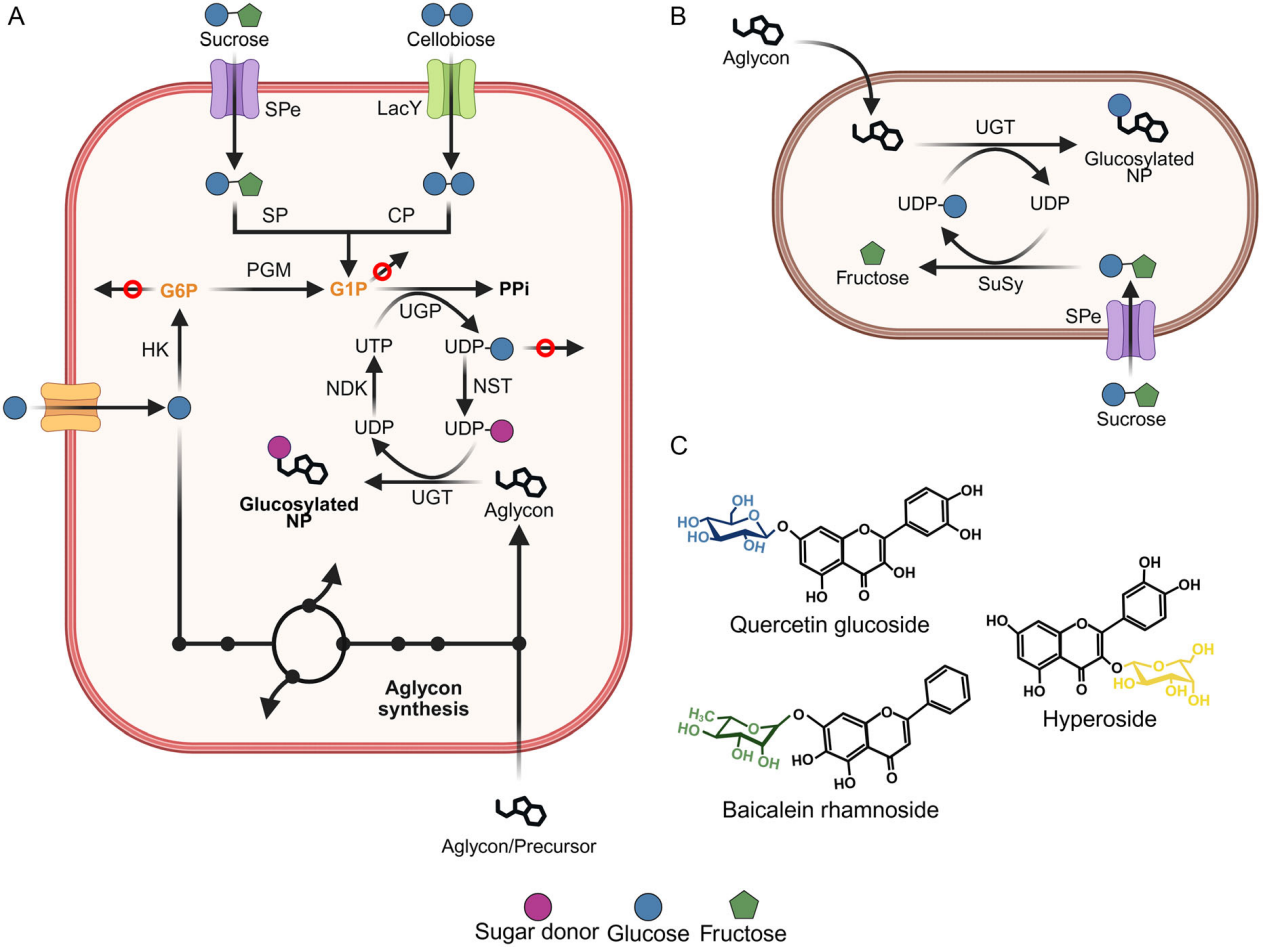
In vivo glycosylation of NPs using whole‐cell biocatalysis (aglycon added to the media) or de novo production (aglycon synthesized from a carbon source or a precursor). A) Carbon sources such as glucose, sucrose, and cellobiose are transported inside the cell^[^ ^116^, ^117^ ^]^ and converted to precursor glucose‐1‐phosphate (G1P) using hexokinase (HK), phosphoglucomutase (PGM), sucrose phosphorylase (SP), or cellobiose phosphorylase (CP).^[^ ^114^, ^116^ ^]^ UDP‐Glc recycling is achieved through UGT, Nucleoside‐diphosphate kinase (NDK), and UDP‐glucose pyrophosphorylase (UGP) with G1P as a key intermediate. UDP‐Glc can also be further converted to other UDP‐sugars in a nucleotide sugar transformation module (NST).^[^ ^84^, ^117^ ^]^ B) Whole‐cell glycosylation using UGT‐SuSy cascade and a sucrose permease (SPe).^[^ ^114^, ^120^ ^]^ C) Examples of in vivo glycosylated NPs, highlighting the diversity of sugar moieties such as glucose, galactose, and rhamnose.^[^ ^114^, ^117^, ^172^ ^]^ Arrows with red circles indicate the knockdown of genes of competing pathways. LacY: lactose permease, G6P: glucose‐6‐phosphate, and UTP: uridine triphosphate.

In addition to in vitro glycosylation reactions, whole‐cell biocatalysis presents an alternative biocatalytic method, which provides the reaction with an intracellular environment that supports enzyme stability and cofactor regeneration (**Figure** **6**).^[^ ^112^ ^]^ Furthermore, the reaction costs can be substantially reduced by avoiding cell lysis and protein purification and by facilitating product recovery via simple reaction media such as water or buffer.^[^ ^112^ ^]^ Despite these advantages, substrate toxicity, protein expression, substrate transport, off‐target reactions inside the cell, and nucleoside diphosphate (NDP)‐sugar regeneration often hinder its implementation. Hence, strategies such as downregulation of genes involved in autolysis from substrate toxicity,^[^ ^113^ ^]^ co‐expression of chaperones,^[^ ^114^ ^]^ and use of surfactants to improve substrate transport^[^ ^115^ ^]^ have been employed and show significant increases in the yields and titers of whole‐cell glycosylation processes.

Increasing and maintaining the intracellular pool of NDP‐sugars is directly correlated with higher glycosylation yields.^[^ ^116^, ^117^, ^118^, ^119^, ^120^, ^121^, ^122^ ^]^ This approach consists of directing the carbon flux from glucose or other carbon sources, such as sucrose or cellobiose, toward the production of UDP‐Glc (Figure 6A).^[^ ^116^, ^117^, ^118^, ^119^, ^120^, ^121^ ^]^ This is achieved by overexpressing genes in *E. coli* such as *pgm* and *ugp*, which encode a phosphoglucomutase and UDP‐glucose pyrophosphorylase, respectively (Figure 6A).^[^ ^116^, ^118^ ^]^ In parallel, knockout of genes involved in competing pathways has also been shown to boost the in vivo glycosylation of NPs. Improved yields have been shown as a result of the knockout of *zwf* and *pgi* to prevent undesired utilization of glucose‐6‐phosphate (G6P),^[^ ^116^, ^118^ ^]^ *agp* and *gcd* to avoid consumption of glucose‐1‐phosphate,^[^ ^118^, ^120^ ^]^ and *ushA*, *ugd* and *otsA* to block use of UDP‐Glc.^[^ ^117^, ^118^, ^120^ ^]^

When UGT‐SuSy cascades are employed (Figure 6B), less gene regulation for maintaining the NDP‐sugar pool is needed, and only the overexpression of genes encoding sucrose transporters has been reported.^[^ ^116^, ^123^ ^]^ Remarkably, SuSys from *Gossypium schwendimani* (*Gs*SuSy) and *Vigna radiata* (*mb*SuSy) have been utilized in these cascades and displayed better activities than conventional SuSys in vivo.^[^ ^115^, ^124^ ^]^ Finally, these different strategies for controlling UDP‐Glc in vivo can be tailored to target glycosylated NPs with diverse sugar moieties such as rhamnosides^[^ ^117^ ^]^ and galactosides^[^ ^114^ ^]^ (Figure 6C).

Recent studies on de novo synthesis of glycosylated NPs have focused on the optimization of both the aglycon production and glycosylation modules (Figure 6A). Similar to whole‐cell biocatalysis, the glycosylation module is based on UGT overexpression and the regulation of pathways competing for UDP‐Glc and its precursors.^[^ ^125^, ^126^, ^127^, ^128^, ^129^, ^130^ ^]^ This pathway regulation can decrease cell viability and growth, ultimately decreasing product synthesis. Separating the precursor production and glycosylation modules can alleviate negative cell responses by allowing for in situ regeneration of UDP‐Glc without compromising cell growth.^[^ ^131^ ^]^ Other bottlenecks, such as product degradation and unspecific precursor glycosylation, have been addressed by downregulating genes encoding glycosidases^[^ ^126^, ^132^, ^133^ ^]^ and using alternative intermediates that cannot be glycosylated by the implemented UGT.^[^ ^134^, ^135^ ^]^ For instance, by disrupting the expression of glycosidases, the production of betanin was improved by 3.6‐fold, a key improvement for the development of more sustainable food colorants.^[^ ^132^ ^]^

### 2.4 Novel Sustainability Improvement Approaches

In addition to the novel chemical and biocatalytic NP glycosylation reactions, there are approaches aimed specifically at reducing environmental impacts and economic costs. A major theme is enzyme immobilization to extend their lifetime in an industrial setting, including covalent linkage to polymer microgels,^[^ ^136^ ^]^ adsorption to metal organic frameworks and magnetic nanospheres,^[^ ^137^, ^138^, ^139^ ^]^ and entrapment in gelatin matrices.^[^ ^140^ ^]^ An analogous strategy for chemical glycosylation is the spatial entrapment of the donor–acceptor pair inside supramolecular capsules.^[^ ^141^ ^]^ The ability to reuse and recycle catalysts will lower the waste and environmental impacts associated with both chemical and biocatalytic approaches, but a sustainability assessment should be performed to ensure that the added benefits outweigh the extra process steps.

Other strategies involve creative utilization of the coupled UGT‐SuSy system for optimizing reaction conditions, including their co‐expression,^[^ ^142^ ^]^ co‐immobilization,^[^ ^143^, ^144^, ^145^, ^146^ ^]^ and direct fusion.^[^ ^147^ ^]^ One particularly interesting system immobilized the UGT, SuSy, and UDP to create a completely self‐sufficient biocatalyst.^[^ ^148^ ^]^ However, the low atom economy and product inhibition of SuSy a known downsides of its use in UDP‐Glc recycling. A more atom‐economical approach replaces SuSy with a four‐enzyme cascade for UDP‐Glc regeneration from maltodextrin and polyphosphate.^[^ ^149^ ^]^ The implementation of more robust enzymes can also improve the efficiency of existing biocatalytic processes, but it is important to consider how these enzyme discovery and engineering campaigns are performed. Advancements to reduce the reagent consumption and time of these campaigns have emerged, including in silico predictors for UGT candidates,^[^ ^150^, ^151^ ^]^ cell‐free enzyme screening platforms,^[^ ^152^ ^]^ high‐throughput screening (HTS) methods for rapid identification of beneficial mutations,^[^ ^153^ ^]^ and HTS methods for identifying enzyme:substrate pairs.^[^ ^154^, ^155^ ^]^ Ultimately, these advancements can assist with the development of more robust and efficient biocatalytic processes with possibly minimized environmental impacts.

## 3 Comparison of Techno‐Economic and Environmental Metrics

The recent advances reviewed here show impressive improvements in many regards compared to previous glycosylation methodologies. Due to the inherent differences between the chemical and biocatalytic methods, each presents different challenges to overcome. In this regard, the general focus of the new chemical methodologies is to improve the regio and stereoselectivities and expand the scope of catalytic approaches for the synthesis of more challenging glycosides. Many of these papers show remarkable improvements, and the donor and acceptor scopes of these approaches are quite vast. In particular, these chemical reactions are generally applied to more rare and non‐natural sugars than their biocatalytic counterparts; however, many of them have not been applied to the glycosylation of NPs. Additionally, the increased stereochemical control of these new methods can be generally assumed to result in a reduction of purification steps and waste generation, suggesting that these methodologies may be superior to their predecessors. On the other hand, since enzymes have inherent regio and stereoselectivities, this is not a focus in the development of new biocatalytic methodologies. Rather, the focus of these studies is the expansion of the donor and acceptor scopes and improving the robustness and efficiencies of these systems. The resulting improvements in titers are impressive, and unlike in the chemical methods, these papers all demonstrate the utility of these systems for the glycosylation of NPs. While the process improvements will certainly reduce waste generation and purification processes compared to wildtype enzymes and unoptimized processes, the scale of this reduction is unknown.

During the review process, we noticed that despite the claims of sustainability and the development of green processes that most of these papers make, very few of these studies include any quantification of green chemistry metrics or perform any life cycle assessment (LCA)‐based analyses. In the chemical glycosylation studies, only one paper actually quantifies the green chemistry metrics of the process, although no comparison to the other syntheses is made.^[^ ^35^ ^]^ Similarly, one paper performs an LCA on the chemical glycosylation of genistein but only compares between the glycosyl derivatives rather than between different syntheses.^[^ ^156^ ^]^ These new chemical methods for NP glycosylation, especially those that do not require protecting and deprotecting steps, provide attractive alternatives to existing methodologies. However, the complete lack of quantification of their environmental impacts leaves uncertainty as to whether these methods are able to outperform their biocatalytic counterparts. Similarly, most of the biocatalytic studies boast lower environmental impacts without any quantifiable metrics to back up such claims. Only a few studies have applied LCAs to compare biocatalytic and conventional approaches for producing glycosylated NPs.^[^ ^93^, ^132^ ^]^ One example assessed the environmental performance of in vivo betanin biosynthesis in a cell factory compared to traditional plant extraction, revealing a 5x reduction in human health and 3x reductions in resource scarcity and ecosystem quality.^[^ ^132^ ^]^ However, in most cases, this lack of quantification prevents any clear conclusions regarding the environmental impacts of these processes and leads us to perform our own techno‐economic and environmental metric quantifications.

**Figure 7:**
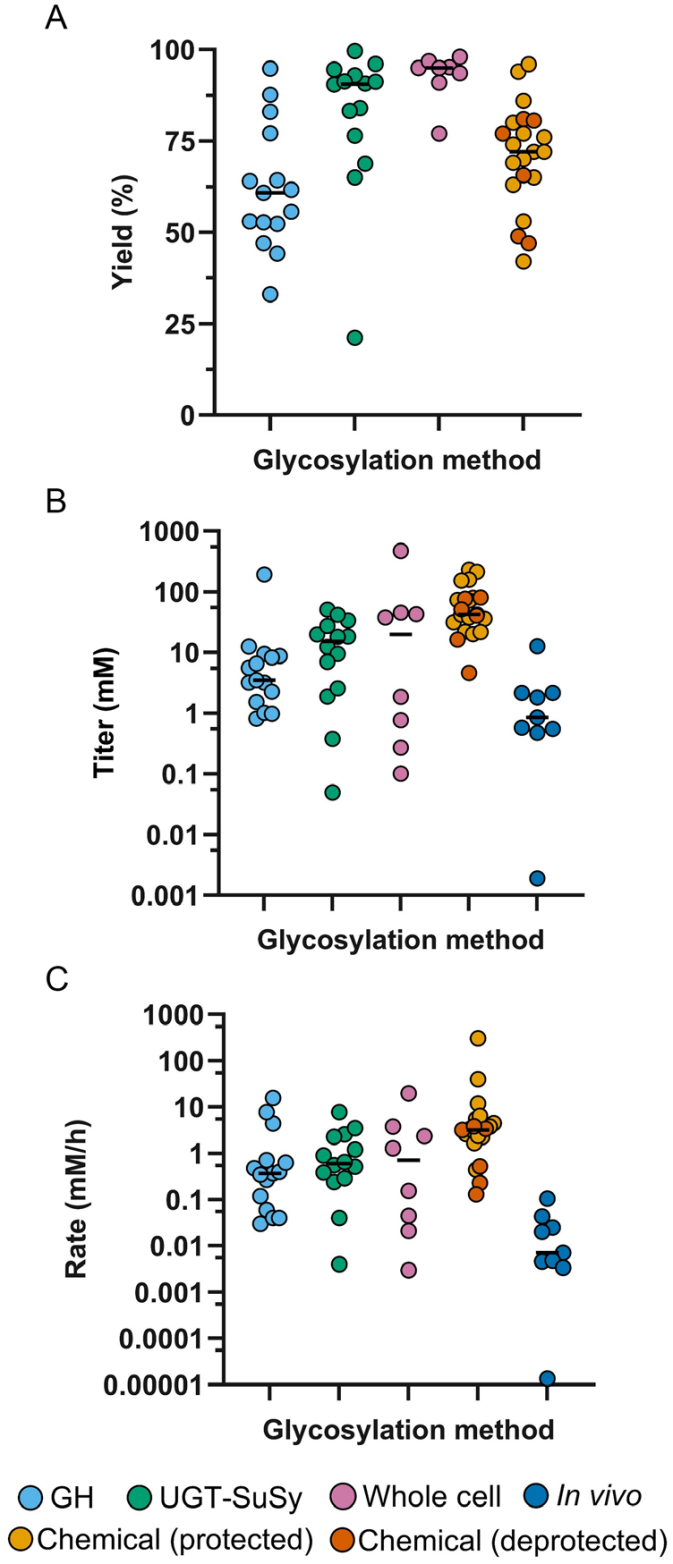
Techno‐economic metrics. A) Reaction conversion yields. B) Reaction titers in log‐scale. C) Reaction rates in log‐scale. Nonisolated yields were plotted if available; otherwise, isolated product yields were plotted (see supplementary). The median of each group is shown with a horizontal bar. The data set is described in Table S1–S5, Supporting Information.

**Figure 8:**
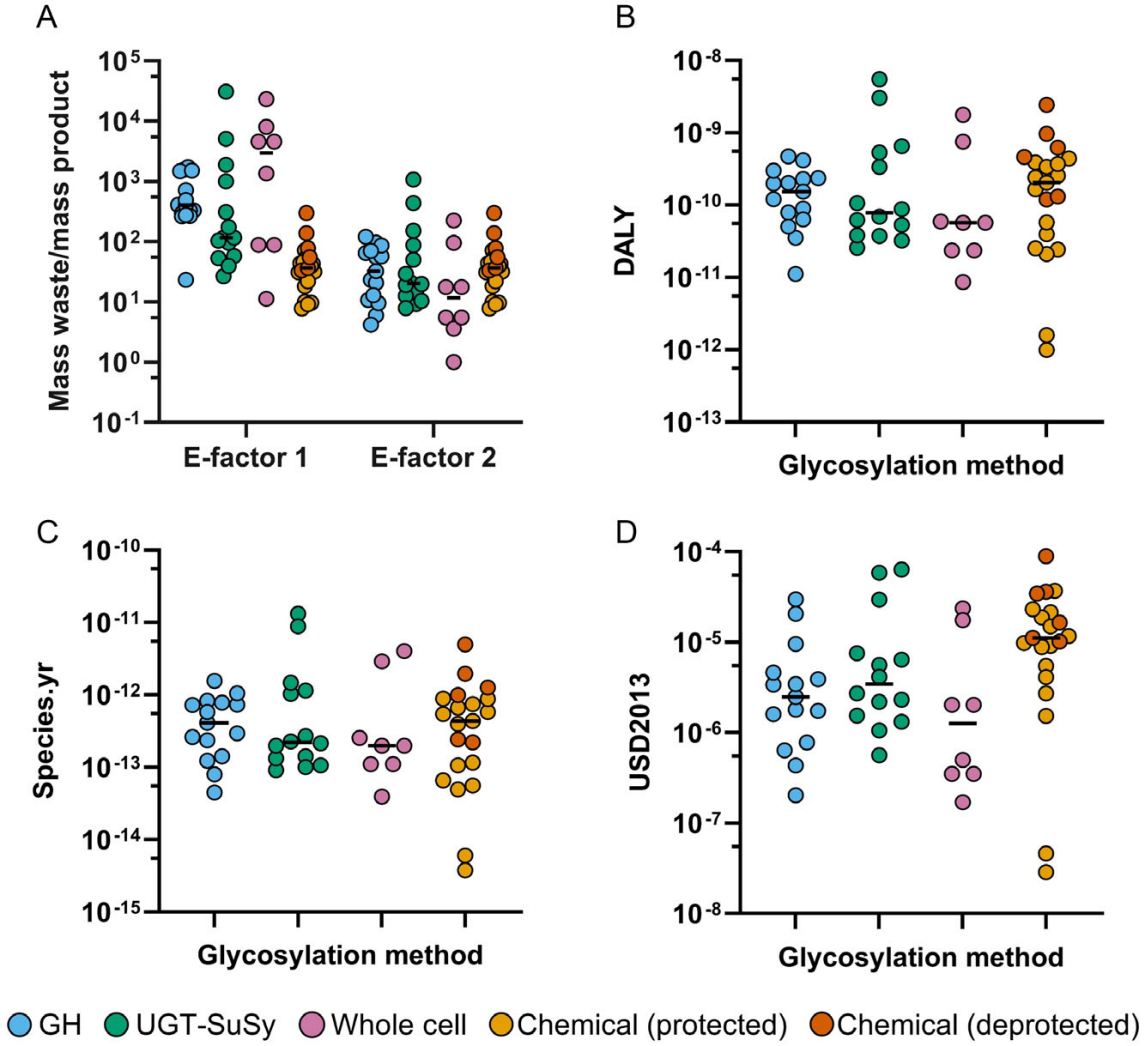
Environmental metrics. A) E‐factors in log‐scale, where E‐factor 1 includes all waste and E‐factor 2 excludes water as waste. B) Human toxicity endpoint with DALY/mg of product in log‐scale. C) Ecosystem quality endpoint with species.yr/mg of product units on log scale. D) Resource scarcity endpoint with USD2013/mg of product units in log‐scale. The median of each group is shown with a horizontal bar. The data set, along with the flows, markets, and allocations, is described in Table S6–S11, Supporting Information.

To compare recent NP glycosylation methods from the last three years, we quantified key metrics such as yield, titer, and rate to estimate their techno‐economic feasibility (**Figure** **7**). Additionally, their environmental performance was assessed (**Figure** **8**) through the calculation of E‐factors and long‐term impacts expressed as the three endpoint impact categories using the ReCiPe 2016 Endpoint (H) impact assessment method, namely ecosystem quality (species.yr^−1^), human health (DALY), and resource scarcity (USD2013). This integrated approach provides a comprehensive understanding of the advantages and limitations of each method, highlighting specific challenges that need to be addressed. A representative NP from each of the reviewed papers was selected for these analyses, and any papers that did not glycosylate any NPs were excluded from this part of the review. As a side note, most of the chemical methods utilized protected sugar donors and did not perform the deprotection as part of the report. However, for those who performed the deprotection chemistry, two analyses were performed: one on the protected compound and one on the deprotected compound, including reagents used in the deprotection steps (shown in dark orange).

### 3.1 Techno‐Economic Evaluation

Techno‐economic metrics evaluate the productivity (rate), efficiency (yield), and scalability (titer) of a process, factors that are closely correlated with its overall economic feasibility. When comparing these metrics (Table 1) within the different glycosylation approaches, we observed that in vitro UGT‐SuSy cascades and whole‐cell biocatalysis exhibited ≈20% and 35% higher yields compared to chemical and in vitro GHs methods, respectively (Figure 7A). The low yields from the chemical methods are a result of downstream purification losses, since isolated yields are typically reported in these papers, as well as the research emphasis on broad substrate specificity rather than optimization for a particular NP, unlike the biocatalytic methods. Furthermore, the majority of studies on whole‐cell biocatalysis employed UGTs,^[^ ^114^, ^116^, ^120^, ^124^, ^157^, ^158^ ^]^ with only a single study utilizing GHs,^[^ ^113^ ^]^ reinforcing the high efficiency of UGTs in catalyzing NP glycosylation. It is important to note that yields can serve as a reference for catalytic efficiency; however, these values are also influenced by the specific nature of the compound being glycosylated and the enzyme/catalyst used.

A different trend is observed when comparing titers and rates, where chemical methods outperformed the biocatalytic methods by ≈3–15x for titer and 5–9x for rate, excluding de novo systems (Figure 7B,C). The limited stability of in vitro enzymes and the mass transfer limitations alongside cellular toxicity in whole‐cell biocatalysis are likely key contributors to these lower titers and rates. Additionally, de novo production of glycosylated NPs showed the lowest titers and rates among the systems analyzed. This outcome can be attributed to the distinct challenges faced by de novo systems, where the cell addresses both the synthesis of the aglycon from a carbon source and also efficiently catalyzes the glycosylation reaction within the intracellular environment.

Considering the economic feasibility of biocatalytic methods, significant advances in enzyme discovery and engineering, together with reaction optimization, have made it possible for UGT‐SuSy cascades to reach high enough yields for feasible industrial implementation^[^ ^14^ ^]^ with median yields of 91% and 95% for in vitro and whole‐cell systems, respectively (Figure 7). However, titers and rates are still limited with only a few examples^[^ ^85^, ^86^, ^87^, ^159^ ^]^ meeting the industrial benchmark^[^ ^14^ ^]^ of 10–50 g.L^−1^ for titer and 1–10 g.L^−1^ h^−1^ for rate, assuming they are high‐priced molecules. Additionally, the absence of reported catalyst usage prevents the calculation of specific yield, a critical metric for determining economic feasibility, given the high cost of enzymes. To improve industrial viability, further enzyme engineering efforts are required to enhance enzyme robustness to achieve the needed titers and rates prior scaling up.

### 3.2 Endpoint Environmental Impact Assessment

Although techno‐economic metrics are commonly used to assess overall performance, they do not account for the environmental impacts associated with a process. The E‐factor has been widely employed as a metric to account for these impacts, as it accounts for the waste generated per mass of product; however, it can be discussed whether the E‐factor properly captures the environmental performance of biosolutions.^[^ ^160^ ^]^ The E‐factor treats all waste as equivalent and, therefore, may not fully capture the environmental performance of biological processes. In this context, biocatalytic methods presented higher E‐factors (E‐factor 1), including whole‐cell systems with the highest values due to low titers (Figure 8A). Generally, the high amount of waste generated in biocatalytic methods is directly linked to the significant amounts of water and excess sucrose required in the process. In contrast, recent chemical methods exhibited lower E‐factors since most of the components are used in stoichiometric amounts and very little catalyst is required. The reaction solvents account for most of the waste in these processes, but are still used in significantly lower quantities than the water in biocatalytic processes. However, it is important to note that the calculated E‐factors generally do not account for activation and protection/deprotection steps and are calculated solely based on the glycosylation reaction (shown in light orange). Reactions that performed the deprotection chemistry (or developed a method that could be performed on an unprotected donor) are shown in dark orange. Additionally, the amount of enzymes in the in vitro methods was not included in the calculation due to insufficient data reported in the biocatalytic methods. When water is excluded (E‐factor 2), biocatalytic methods display comparable or even lower E‐factors than chemical methods (Figure 8A). This is particularly evident in whole‐cell biocatalysis, resulting in the lowest E‐factors of all the processes, highlighting the significant contribution of water consumption to the E‐factor of biocatalytic processes.

Given the known limitations of the E‐factor, the long‐term impacts of glycosylation reactions on ecosystem quality, human health, and resource scarcity were quantified using a mass‐based impact assessment approach.^[^ ^161^ ^]^ However, not all components were available in the ecoinvent v3.8 database (Table S6, Supporting Information), making this the best possible approximation of these processes given the available information. The impact of the chemical methods was higher than that of the biocatalytic processes, with impacts 1–4x, 1‐2x, and 3–9x higher for human toxicity, ecosystem quality, and resource scarcity, respectively (Figure 8B–D). These differences are attributed to the toxicity and resource‐intensive nature of the compounds used in chemical glycosylation. UDP and UDP‐Glc were not accounted for (since they were not in the database), which may influence the interpretation of the results of the UGT‐SuSy cascade. However, most reactions were carried out using cell lysates of co‐expressed UGT and SuSy without the addition of exogenous UDP, which might represent one of the most sustainable setups for biocatalytic glycosylation.^[^ ^85^, ^86^, ^87^, ^95^, ^159^, ^162^, ^163^, ^164^ ^]^ Similarly, whole‐cell systems exhibited the lowest impacts across the three endpoint categories, attributed to minimal input requirements plus additional potential reductions due to intracellular UDP/UDP‐Glc production. These endpoints better capture the impacts of the chemical processes compared to the enzymatic ones, taking into account that all chemical methods use critical elements that will be depleted in the next 5–500 years (Table S15, Supporting Information), and that the waste generated is more environmentally toxic (Table S16, Supporting Information).^[^ ^15^ ^]^ For this analysis, waste was classified as environmentally hazardous if it was identified with H400, H401, H410, H411, H412, H413, and H420 hazard categories on the PubChem database (compounds that were not available in the PubChem database were not considered). Of the compounds used and in the PubChem database, we identified a number of compounds with Hazard Statements H411, H412, and H413. The majority of the environmentally hazardous compounds used are catalysts containing Pd (Pd(PPh_3_)_4_, Pd_2_(dba)_3_, Pd(PhCN)_2_Cl_2_) and fluorinated compounds (trifluoroacetic acid (TFA), C_4_F_9_I). Importantly, the media used for enzyme or cell synthesis in biocatalytic processes and the activation and deprotection steps in chemical methods were not accounted for in this assessment, and a complete comparative LCA should be developed to further model these processes. For instance, for chemical methods that included deprotection steps (dark orange data points), impacts increased 6–20x across all three impact categories compared to the best biocatalytic process (whole‐cell systems) (Figure 8B–D). However, the environmental performance of these systems could improve if solvent recycling, catalyst regeneration, and industrial process setups are implemented.^[^ ^165^ ^]^

The reaction conditions play a key role in the sustainability of the process. Particularly, solvent choice is critical in in vitro glycosylation reactions, as it can both enhance conversion yields and drive protein denaturation, which could significantly contribute to the impact on all three endpoint categories (Table S12–S14, Supporting Information). Unfortunately, studies investigating solvent effects on biocatalytic systems are limited, making it difficult to discern the specific solvent impact on each enzyme. Furthermore, most research on biocatalytic systems has been done using DMSO, and few studies have assessed the use of alternative solvents. One study demonstrated that using methanol yielded similar conversions to DMSO, with the added benefit of easier recovery.^[^ ^166^ ^]^ Additionally, several studies have shown the benefit of adding 2‐hydroxypropyl‐β‐cyclodextrin for aglycon encapsulation, improving its solubility and ultimately leading to higher yields and reaction rates, showcasing the need for research into alternative reaction media for biocatalytic glycosylation reactions.^[^ ^166^, ^167^, ^168^ ^]^

Sucrose is another major contributor to the impacts associated with the biocatalytic methods (Table S12–S14, Supporting Information), and maximizing its utilization should be a key focus for future research. Converting the fructose waste from SuSy and sucrose phosphorylase reactions into a high‐value product, such as D‐allulose, can increase the reaction efficiency by driving the equilibrium toward the production of UDP‐Glc, enhancing the reaction yield, and improving the atom economy by eliminating fructose as a byproduct.^[^ ^123^, ^169^ ^]^ Additionally, the fructose can also be used for growth, which represents a more carbon‐efficient approach, avoiding the use of an extra carbon source such as glycerol or glucose for UDP‐Glc production inside the cell, and promotes the implementation of a more sustainable strategy for the glycosylation of NPs.

## 4 Summary and Outlook

The glycosylation of NPs remains a critical strategy to enhance properties such as stability, solubility, and bioavailability, thereby broadening product applicability and process feasibility. Recent advancements have focused on both biocatalytic and chemical glycosylation methods, achieving relatively high yields but often limited by titers and reaction rates. Chemical methods have increasingly emphasized eliminating and minimizing protecting steps, while biocatalytic approaches have prioritized reaction optimization and the engineering of robust enzymes with enhanced catalytic efficiencies. Significant differences between these methods emerge from techno‐economic considerations. Although GHs do not require activated sugar donors and engineered versions tackled competing activities, yields are still not competitive compared to UGT‐SuSy cascades, or they require expensive sugar donors such as α‐D‐glucopyranosyl fluoride. However, there are a few cases where GHs stand out; for instance, the use of branching sucrases avoids undesired sugar oligomerization, and sucrose phosphorylase was used in an integrated process for glycerol transglycosylation, where the fructose by‐product and the excess of glycerol were valorized.^[^ ^108^, ^170^ ^]^ Additionally, GHs may represent a viable option when the length of the glycoside chain attached to the acceptor does not compromise product purification, activity, or final application.

Our analysis suggests a number of process considerations to use as a framework for understanding and addressing the bottlenecks of glycosylation reactions. To further improve the environmental performance of UGT‐SuSy‐based systems, whole‐cell biocatalysis can be utilized, leveraging intracellular UDP/UDP‐Glc pools. These approaches offer benefits such as improved enzyme stability and simplified product purification, but their reliance on high quantities of sucrose remains a key challenge. In this context, valorization of fructose release from the reaction and product purification strategies for sucrose recycling should be employed. The role of solvents in biocatalytic systems is also critical, as they aid substrate solubilization while significantly contributing to the environmental impact. Consequently, the development of robust enzymes capable of functioning in more sustainable solvents should become a central focus of research. Recent research exploring the in vivo production of glycosylated NPs shows promise for sustainability, as it allows for the biosynthesis of glycosylated products while maximizing carbon utilization. However, achieving the yields and titers necessary to compete with existing industrial processes remains a significant challenge.

In summary, we found that the majority of recent reports on NP glycosylation claim to lower the environmental impacts without providing any quantitative assessment. Additionally, the lack of consistent and thorough reporting of experimental conditions in the biocatalytic research articles hindered the depth and quality of the analysis we were able to perform. There is an urgent need in the biocatalysis community to change publication specifications, and Standards for Reporting Enzymology Data (STRENDA) provides an excellent starting point.^[^ ^171^ ^]^ While this study provides actionable recommendations for improving glycosylation in terms of sustainability, a comprehensive analysis within the LCA framework is necessary to fully understand the environmental impacts of these systems. This should include evaluations of advanced strategies such as enzyme immobilization, enzyme co‐expression, and whole‐cell biocatalysis to determine their true contributions to sustainable glycosylation reactions. Despite the uncertainty of environmental quantitative frameworks, they provide the closest approximation to the real effects, and research should include these metrics to reduce greenwashing in the biocatalytic field. Our analysis shows that new glycosylation processes cannot claim to be more sustainable without quantitative evidence (i.e., LCA) to back up these claims.

## Supporting Information

The authors have cited additional references within the Supporting Information.^[170–172]^

A supplementary excel file is included which contains all data extracted from the literature and calculationsperformed for our analyses.

## Conflict of Interest

The authors declare no conflict of interest.

## Supporting information

Supplementary Material
