# Supplementary material for: Sustainable Natural Product Glycosylation: A Critical Evaluation of Biocatalytic and Chemical Approaches
Source: ChemSusChem. 2025 Sep 15;18(20):e202501094. doi: 10.1002/cssc.202501094 (PMC12548952; doi:10.1002/cssc.202501094)
Supplement: Supplementary file 1 — Supplementary Material [file CSSC-18-e202501094-s001.zip › cssc202501094-sup-0001-SuppData-S1.pdf]

Supplementary Information

Sustainable natural product glycosylation: a critical evaluation of biocatalytic and chemical approaches

Felipe Mejia-Otalvaro[a§], Brianna Marie Lax[a§], Onur Kırtel[a], Ditte Hededam Welner[a*]

[a] The Novo Nordisk Center for Biosustainability, Technical University of Denmark, Søltofts Plads, Building 220, Kgs. Lyngby DK-2800, Denmark
[§] These authors contributed equally to this work
[*] Corresponding author, diwel@biosustain.dtu.dk, +45 93 51 34 98
Author emails (in order): felmeo@biosustain.dtu.dk; blax@biosustain.dtu.dk; okirtel@biosustain.dtu.dk

Supporting Tables

1. The values plotted in Figure 2 are listed in Tables S1 to S5.

2. Table S6 and S7 lists the impact on the three endpoint categories of 1 mg of the components found in ecoinvent v3.8 database and that were considered in the calculation of the impact for all the glycosylation methods based on a mass balance.

3. The values plotted in Figure 3 are listed in Tables S8 to S11.

4. The contribution to the environmental impacts of the reaction components for the biocatalytic methods are listed in Tables S12 to S14.

5. The critical elements employed in the chemical methods are listed in Table S15.

6. The hazardous compounds used in the chemical methods are listed in Table S16.

Table S1. Techno-economic metrics for *in vitro* glycosyl hydrolases.

Product	Conversion yield (%)	Titer (mM)	Rate (mM h^{-1})	Reference
Myricetin α-triglucoside	64.00	3.20	0.27	[101]
Daidzein α-D-glucosyl derivatives	87.70	8.77	0.37	[102]
Genistein α-D-glucosyl derivatives	94.90	9.49	0.40	[102]
Glycitein α-D-glucosyl derivatives	77.10	1.54	0.06	[102]
Naringenin-α-maltose	56.00	5.60	0.12	[103]
Ganoderic acid F glycosides	47.00	0.82	0.03	[104]
Ganoderic acid A glycoside	53.00	1.02	0.04	[104]
Ganoderic acid G glycoside	52.40	0.98	0.04	[104]
Ethyl vanillin-α-glucoside	52.80	12.58	15.73	[108]
Genistein 7,4′-O-α-diglucoside	33.00	6.60	4.40	[109]
Epigallocatechin gallate glucoside	44.20	2.27	0.48	[110]
Epigallocatechin gallate sophoroside	64.30	3.22	0.64	[110]
Epigallocatechin gallate xyloside	61.70	3.52	0.72	[110]
α-Arbutin	60.90	193.30	7.73	[111]
(+)-Catechin-3'-O-α-D-glucopyranoside	83.00	8.30	0.35	[107]

Table S2. Techno-economic metrics for *in vitro* UGT-SuSy cascade.

Product	Conversion yield (%)	Titer (mM)	Rate (mM h^{-1})	Reference
Glycyrrhetic acid 3-O-mono-b-D-glucuronide	96.10	0.38	0.04	[90]
Quercetin-3,4′-O-diglucoside	21.20	7.00	0.29	[92]
Arabinosylated betunilic acid	83.30	0.05	0.004	[84]
Rebaudioside M	90.50	18.10	2.59	[85]
Rebaudioside D	91.29	18.26	1.22	[86]
Ginsenosides Rg3	68.78	12.38	0.52	[88]
Rebaudioside M	76.48	51.06	0.90	[94]
Neoliquiritin	65.00	2.60	7.81	[95]
Rebaudioside D2	94.66	9.47	0.39	[162]
Mono-β-1,6-glucosylated Rebaudioside A	91.34	27.40	2.28	[159]
Cinnamyl alcohol mono-glucoside (rosin)	90.80	33.50	0.56	[163]
Salidroside	99.60	19.92	0.66	[164]
Cinnamyl alcohol mono-glucoside (rosin)	84.00	42.00	3.50	[166]
Gastrodin	93.00	1.89	0.24	[91]

Table S3. Techno-economic metrics for whole cell reactions.

Product	Conversion yield (%)	Titer (mM)	Rate (mM h^{-1})	Reference
α-Arbutin	95.20	476.03	19.83	[113]
Hyperoside	93.60	1.87	0.156	[114]
Nothofagin	98.00	0.10	0.003	[116]
Rebaudioside D	95.00	42.70	1.29	[124]
Quercetin 7-O-glucoside	77.00	0.27	0.045	[157]
Cinnamyl alcohol mono-glucoside (rosin)	91.00	45.50	3.792	[166]
Gastrodin	95.00	0.77	0.021	[91]
Siamenoside	96.90	37.80	2.36	[122]

Table S4. Techno-economic metrics for *de novo* synthesis.

Product	Titer (mM)	Rate (mM h^{-1})	Reference
Ginsenoside F2	0.478	0.0034	[126]
Rubusosides	2.178	0.020	[127]
Rosavin E	1.82	0.025	[128]
Ginsenoside Ro	0.552	0.0046	[129]
Glycyrrhetinic Acid 3-O-monoglucuronide	0.853	0.0071	[130]
Glycyrrhizin	0.578	0.0048	[130]
Salidroside	12.654	0.105	[131]
Betanin	2.1798	0.043	[132]
Glycosylated astaxanthin	0.0019	0.000013	[134]

Table S5. Techno-economic metrics for chemical methods.

Product	Conversion yield (%)	Titer (mM)	Rate (mM h^{-1})	Reference
Stanolone derivative glycoside (deprotected)	81.00	81.00	3.375	[53]
Estrone derivative glycoside (deprotected)	77.00	77.00	3.208	[53]
Epiandrosterone fucoside derivative (protected)	86.00	233.50	311.333	[34]
Menthol glycoside derivative (protected)	63.00	31.50	2.250	[61]
Rutaecarpine derivative mannoside (protected)	53.00	22.08	5.521	[54]
D-glucal-D-mannofuranose-estrone (protected)	42.00	42.00	2.625	[67]
Lactone precursor glycoside (protected)	80.00	80.00	1.667	[56]
Lactone glycoside (deprotected)	65.60	16.40	0.228	[56]
Capsaicin glucoside (protected)	74.00	74.00	3.083	[63]
Simvastatin glycoside (protected)	94.00	20.14	3.357	[55]
Ibuprofen derivative glycoside (protected)	72.00	72.00	4.500	[30]
Naproxen derivative glycoside (protected)	76.00	38.00	3.800	[68]
Cholesterol 6-O-myristoyl glucoside (protected)	70.00	215.75	11.986	[52]
Cholesterol glucoside (protected)	72.00	36.00	3.000	[36]
Tropolone glucoside (protected)	96.00	160.00	40.000	[66]
Tropolone glucoside (deprotected)	80.64	40.32	0.531	[66]
Oleandrigenin glucoside (protected)	96.00	160.00	40.000	[33]
Oleandrigenin glucoside (deprotected)	48.99	4.66	0.129	[33]
Empagliflozin galactoside (protected)	65.00	21.67	0.451	[42]
Naringenin-7-O-D-glucopyranoside (prunin) (deprotected)	47.00	51.70	3.830	[31]
Naproxen glycoside (protected)	77.00	154.00	6.417	[40]

Table S6. Impact on endpoint categories of 1 mg of reagents and compounds used in the reactions and found in the ecoinvent v3.8 database. The ReCiPe 2016 Endpoint (H) impact assessment method was used.

Compound	Human health (DALY)	Ecosystem quality (species.yr)	Resources scarcity (USD2013)
DMSO	3.89249E-12	7.04908E-15	3.37118E-07
Sodium phosphate	1.45432E-11	2.14317E-14	1.65127E-07
Glucose	3.52004E-12	1.33946E-14	1.02416E-07
Sucrose	2.36166E-12	1.20116E-14	5.01933E-08
Deionized water	2.53794E-15	1.09409E-17	2.13575E-11
HOAc	4.07255E-12	7.76161E-15	3.56171E-07
Acetonitrile	9.0252E-12	2.09779E-14	9.03575E-07
DCM	8.10965E-12	1.61194E-14	2.15198E-07
Dioxane	1.00078E-11	2.13018E-14	7.48974E-07
EtOAc	6.64572E-12	1.32927E-14	5.35619E-07
$LiClO_4$	2.671E-11	4.71217E-14	7.00924E-07
MTBE	2.14364E-12	4.68076E-15	5.1504E-07
DMF	6.5525E-12	1.30558E-14	5.38165E-07
K_2CO_3	8.22176E-12	1.43112E-14	2.13207E-07
$K_2S_2O_8$	3.8854E-12	6.59441E-15	1.1311E-07
Styrene	6.53745E-12	1.37073E-14	7.1642E-07
THF	1.51308E-11	3.11414E-14	6.38811E-07
Toluene	2.33693E-12	6.15823E-15	5.60478E-07
HCl	1.95011E-12	3.4444E-15	4.52603E-08

Table S7. Flow, market, and allocation of the compounds used in Table S6.

Compound	Flow	Market
DMSO	Dimethyl sulfoxide	Market for dimethyl sulfoxide | dimethyl sulfoxide | APOS, S - GLO
Sodium phosphate	Sodium phosphate	Market for sodium phosphate | sodium phosphate | APOS, S - RER
Glucose	Glucose	Market for glucose | glucose | APOS, S - GLO
Sucrose	Sugar, from sugar beet	Market for sugar, from sugar beet | sugar, from sugar beet | APOS, S - GLO
Deionized water	Water, deionised	Market for water, deionised | water, deionised | APOS, S - Europe without Switzerland
HOAc	Acetic acid, without water, in 98% solution state	Market for acetic acid, without water, in 98% solution state | acetic acid, without water, in 98% solution state | APOS, S - GLO
Acetonitrile	Acetonitrile	Market for acetonitrile | acetonitrile | APOS, S - GLO
DCM	Dichloromethane	Market for dichloromethane | dichloromethane | APOS, S - RER
Dioxane	Dioxane	Market for dioxane | dioxane | APOS, S - RER
EtOAc	Ethyl acetate	Market for ethyl acetate | ethyl acetate | APOS, S - GLO
$LiClO_4$	Lithium chloride	Market for lithium chloride | lithium chloride | APOS, S - GLO
MTBE	Methyl tert-butyl ether	Market for methyl tert-butyl ether | methyl tert-butyl ether | APOS, S - GLO
DMF	N,N-dimethylformamide	Market for N,N-dimethylformamide | N,N-dimethylformamide | APOS, S - GLO
K_2CO_3	Potassium carbonate	Market for potassium carbonate | potassium carbonate | APOS, S - GLO
$K_2S_2O_8$	Potassium sulfate	Market for potassium sulfate | potassium sulfate | APOS, S - RER
Styrene	Styrene	Market for styrene | styrene | APOS, S - GLO
THF	Tetrahydrofuran	Market for tetrahydrofuran | tetrahydrofuran | APOS, S - GLO
Toluene	Toluene, liquid	Market for toluene, liquid | toluene, liquid | APOS, S - RER
HCl	Hydrochloric acid, without water, in 30% solution state	Market for hydrochloric acid, without water, in 30% solution state | hydrochloric acid, without water, in 30% solution state | APOS, S - RER

Table S8.E-factors and impact on endpoints of *in vitro* glycosyl hydrolases per mg of glycosylated product.

Product	E-factor 1	E-factor 2	Human health (DALY)	Ecosystem quality (species.yr)	Resources scarcity (USD2013)	Reference
Myricetin α-triglucoside	407.95	97.28	4.17E-10	7.84E-13	2.99E-05	[101]
Genistein α-D-glucosyl derivatives	263.55	20.83	7.94E-11	2.62E-13	1.60E-06	[102]
Daidzein α-D-glucosyl derivatives	296.11	23.37	8.92E-11	2.95E-13	1.80E-06	[102]
Glycitein α-D-glucosyl derivatives	1568.00	121.09	4.73E-10	1.56E-12	9.56E-06	[102]
Naringenin-α-maltoside	331.05	32.56	1.21E-10	4.10E-13	2.48E-06	[103]
Ganoderic acid A glycoside	1492.40	55.96	1.98E-10	7.22E-13	3.39E-06	[104]
Ganoderic acid G glycoside	1520.41	57.06	2.02E-10	7.35E-13	3.45E-06	[104]
Ganoderic acid F glycosides	1721.88	64.64	2.29E-10	8.33E-13	3.91E-06	[104]
Ethyl vanillin-α-glucoside	328.26	86.14	2.37E-10	1.05E-12	4.61E-06	[108]
Genistein 7,4′-O-α-diglucoside	267.79	12.89	1.53E-10	2.36E-13	1.75E-06	[109]
Epigallocatechin gallate sophoroside	403.57	6.00	3.54E-11	7.96E-14	4.35E-07	[110]
Epigallocatechin gallate xyloside	493.46	9.61	5.06E-11	1.23E-13	6.38E-07	[110]
Epigallocatechin gallate glucoside	720.28	10.76	6.31E-11	1.42E-13	7.77E-07	[110]
α-Arbutin	23.22	4.21	1.12E-11	4.49E-14	2.04E-07	[111]
(+)-Catechin-3'-O-α-D-glucopyranoside	278.16	70.37	3.00E-10	5.82E-13	2.07E-05	[107]

E-factor 1 and 2 refer to all the waste generated (enzyme was not considered) and excluding water, respectively.

Table S9. E-factors and impact on endpoints of *in vitro* UGT-SuSy cascade per mg of glycosylated product.

Product	E-factor 1	E-factor 2	Human health (DALY)	Ecosystem quality (species.yr)	Resources scarcity (USD2013)	Reference
Glycyrrhetic acid 3-O-mono-b-D-glucuronide	5096.17	1074.71	3.03E-09	1.33E-11	5.85E-05	[90]
Quercetin-3,4′-O-diglucoside	311.25	151.54	5.33E-10	1.47E-12	2.97E-05	[92]
Arabinosylated betunilic acid	31019.52	439.72	5.49E-09	8.91E-12	6.37E-05	[84]
Rebaudioside M	53.27	12.63	3.74E-11	1.33E-13	1.32E-06	[85]
Rebaudioside D	57.77	14.11	5.32E-11	1.42E-13	2.32E-06	[86]
Ginsenosides Rg3	116.97	29.50	1.06E-10	2.69E-13	6.42E-06	[88]
Rebaudioside M	26.87	9.25	2.57E-11	9.09E-14	1.06E-06	[94]
Neoliquiritin	1006.75	87.49	3.38E-10	1.15E-12	5.60E-06	[95]
Rebaudioside D2	104.06	19.86	8.75E-11	1.98E-13	4.16E-06	[162]
Mono-β-1,6-Glucosylated Rebaudioside A	39.49	10.41	3.25E-11	1.00E-13	1.54E-06	[159]
Cinnamyl alcohol mono-glucoside (rosin)	116.53	20.84	6.89E-11	2.27E-13	2.73E-06	[163]
Salidroside	175.03	7.86	3.81E-11	1.06E-13	5.65E-07	[164]
Cinnamyl alcohol mono-glucoside (rosin)	95.59	19.26	6.21E-11	2.15E-13	2.20E-06	[166]
Gastrodin	1902.41	50.56	6.55E-10	1.03E-12	7.57E-06	[91]

E-factor 1 and 2 refer to all the waste generated (enzyme was not considered) and excluding water, respectively.

Table S10. E-factors and impact on endpoints of whole cell reactions per mg of glycosylated product.

Product	E-factor 1	E-factor 2	Human health (DALY)	Ecosystem quality (species.yr)	Resources scarcity (USD2013)	Reference
α-Arbutin	11.32	3.60	8.63E-12	3.91E-14	1.7E-07	[113]
Hyperoside	1362.74	223.92	7.55E-10	2.91E-12	1.76E-05	[114]
Nothofagin	23383.42	1.01	5.93E-11	2.56E-13	4.99E-07	[116]
Rebaudioside D	88.24	17.78	5.73E-11	1.98E-13	2.03E-06	[124]
Quercetin 7-O-glucoside	8102.96	95.68	1.77E-09	4.04E-12	2.38E-05	[157]
Cinnamyl alcohol mono-glucoside (rosin)	88.24	17.78	5.73E-11	1.98E-13	2.03E-06	[166]
Gastrodin	4601.84	5.51	2.36E-11	1.11E-13	3.52E-07	[91]
Siamenoside	4601.84	5.51	2.36E-11	1.11E-13	3.52E-07	[122]

E-factor 1 and 2 refer to all the waste generated (enzyme was not considered) and excluding water, respectively.

Table S11. E-factor and impact on endpoints of chemical methods.

Product	E-factor 1	Human health (DALY)	Ecosystem quality (species.yr)	Resources scarcity (USD2013)	Reference
Stanolone derivative glycoside (deprotected)	33.30	2.14E-09	4.04E-12	1.87E-04	[53]
Estrone derivative glycoside (deprotected)	36.66	2.18E-09	4.04E-12	1.87E-04	[53]
Epiandrosterone fucoside derivative (protected)	10.08	4.34E-09	1.18E-11	9.78E-04	[34]
Menthol glycoside derivative (protected)	43.66	3.02E-08	6.94E-11	2.89E-03	[61]
Rutaecarpine derivative mannoside (protected)	31.79	1.85E-08	4.45E-11	2.40E-03	[54]
D-glucal-D-mannofuranose-estrone (protected)	31.05	1.04E-08	2.24E-11	7.77E-04	[67]
Lactone precursor glycoside (protected)	30.71	1.50E-08	3.62E-11	1.45E-03	[56]
Lactone glycoside (deprotected)	301.40	1.01E-07	2.08E-10	3.73E-03	[56]
Capsaicin glucoside (protected)	18.38	4.20E-09	1.13E-11	9.74E-04	[63]
Simvastatin glycoside (protected)	71.94	1.95E-09	7.44E-12	5.69E-05	[55]
Ibuprofen derivative glycoside (protected)	21.90	2.16E-08	4.62E-11	1.59E-03	[30]
Naproxen derivative glycoside (protected)	54.75	2.16E-08	4.31E-11	5.74E-04	[68]
Cholesterol 6-O-myristoyl glucoside (protected)	9.75	1.60E-09	3.24E-12	4.26E-05	[52]
Cholesterol glucoside (protected)	43.12	2.20E-08	4.37E-11	5.83E-04	[36]
Tropolone glucoside (protected)	7.79	1.54E-09	4.08E-12	3.00E-04	[66]
Tropolone glucoside (deprotected)	138.80	2.31E-08	4.70E-11	8.72E-04	[66]
Oleandrigenin glucoside (protected)	46.38	4.11E-09	8.29E-12	1.09E-04	[33]
Oleandrigenin glucoside (deprotected)	78.49	4.11E-09	8.29E-12	1.09E-04	[33]
Empagliflozin galactoside (protected)	39.48	6.34E-11	2.41E-13	1.85E-06	[42]
Naringenin-7-O-D-glucopyranoside (prunin) (deprotected)	55.13	1.04E-07	2.23E-10	7.76E-03	[31]
Naproxen glycoside (protected)	9.19	1.90E-09	3.70E-12	2.06E-04	[40]

E-factor 1 refers to all the waste generated.

Table S12. Contribution in percentage of reaction components to the three endpoint categories for glycosyl hydrolases.

Reference	Contribution to human health (%)	Contribution to ecosystem quality (%)	Contribution to resource scarcity (%)

	SD	DMSO	Water	Buffer	SD	DMSO	Water	Buffer	SD	DMSO	Water	Buffer
[101]	4	80	0	16	10	77	0	13	1	96	0	3
[102]	50	5	1	44	76	3	1	20	52	23	0	25
[103]	52	5	1	42	78	3	1	19	54	22	0	24
[104]	59	0	2	40	82	0	2	16	73	0	1	26
[108]	82	0	0	18	94	0	0	6	90	0	0	10
[109]	3	0	0	96	7	0	1	92	4	0	0	96
[110] α-GF	36	0	3	61	64	0	4	32	51	0	2	47
[110] pNP-Xyl	45	0	2	52	64	0	4	32	51	0	2	47
[111]	70	0	0	29	89	0	0	11	82	0	0	18
[107]	6	76	0	18	15	71	0	14	2	95	0	3

SD: sugar donor. Reference 6 uses two conditions depending on the sugar donor: α-GF (alpha-D-Glucopyranosyl fluoride) and pNP-Xyl (4-Nitrophenyl b-D-xylopyranoside).

Table S13. Contribution in percentage of reaction components to the three endpoint categories for the UGT-SuSy cascade.

Reference	Contribution to human health (%)				Contribution to ecosystem quality (%)				Contribution to resource scarcity (%)			
	Sucrose	DMSO	Water	Buffer	Sucrose	DMSO	Water	Buffer	Sucrose	DMSO	Water	Buffer
[90]	80	0	0	19	93	0	0	6	89	0	0	11
[92]	30	55	0	15	56	36	0	8	12	85	0	3
[84]	3	0	1	96	9	0	4	87	5	0	1	93
[85]	56	25	0	20	79	12	0	8	33	60	0	6
[86]	29	39	0	31	56	26	0	17	14	77	0	8
[88]	24	62	0	14	47	44	0	8	8	89	0	3
[94]	54	33	0	13	78	17	0	5	28	69	0	4
[95]	53	0	1	47	79	0	1	20	68	0	0	32
[162]	17	46	0	37	39	37	0	24	8	83	0	9
[159]	40	43	0	17	66	25	0	8	18	78	0	4
[163]	47	31	0	21	73	17	0	9	25	68	0	6
[164]	35	0	1	63	65	0	2	34	51	0	1	49
[166]	52	25	0	22	77	13	0	9	31	61	0	7
[91]	2	0	1	97	7	0	2	91	4	0	1	95

Table S14. Contribution in percentage of reaction components to the three endpoint categories for whole cell reactions.

Reference	Contribution to human health (%)				Contribution to ecosystem quality (%)				Contribution to resource scarcity (%)			
	SD	DMSO	Water	Buffer	SD	DMSO	Water	Buffer	SD	DMSO	Water	Buffer
[113]	84	0	0	15	95	0	0	5	91	0	0	9
[114]	67	7	0	26	86	3	0	10	63	24	0	13
[116]	0	0	100	0	0	0	100	0	0	0	100	0
[124]	58	0	0	41	83	0	0	17	72	0	0	27
[157]	21	0	1	78	48	0	2	50	34	0	1	66
[166]	52	25	0	22	77	13	0	9	31	61	0	7
[91]	51	0	49	0	55	0	45	0	72	0	28	0
[122]	100	0	0	0	100	0	0	0	100	0	0	0

SD: sugar donor.

Table S15. Critical elements used in the chemical methods.

Reference	5-50 year supply	50-100 year supply	100-500 year supply
[53]		P	

[34]	Bi		
[61]	Pt	Li	
[54]		Cu, P	
[67]		Pd, P	I
[56]	W	P, Ni	
[63]		Pd, P, S	I
[55]		S	
[30]	Ru	P	
[68]		Pd, P	
[52]		S	
[36]	Hf	S	
[66]		Pd, P	
[33]		P	
[42]		P, S	
[31]		P	
[40]		P, S	I

Table S16. Environmentally hazardous compounds used in the chemical methods.

Reference	Hazardous Compounds		
	H411	H412	H413
[53]		DABCO	
[67]			$Pd(PhCN)_2Cl_2$
[56]		TFA	
[63]			$Pd(PPh_3)_4$
[55]		Dichloroethane	
[30]		$RuCl_2$(*p*-cymene)	
[68]			$Pd(PPh_3)_4$
[66]	$Pd_2(dba)_3$		
[31]		Ph_3P	
[40]	C_4F_9I	PPh_3O	
